# Understanding the Pathophysiological Actions of Tau Oligomers: A Critical Review of Current Electrophysiological Approaches

**DOI:** 10.3389/fnmol.2020.00155

**Published:** 2020-08-20

**Authors:** Emily Hill, Mark J. Wall, Kevin G. Moffat, Thomas K. Karikari

**Affiliations:** ^1^School of Life Sciences, Gibbet Hill Campus, University of Warwick, Coventry, United Kingdom; ^2^Department of Psychiatry and Neurochemistry, Institute of Neuroscience and Physiology, University of Gothenburg, Gothenburg, Sweden

**Keywords:** tau, oligomer, neuron, electrophysiology, tauopathy

## Abstract

Tau is a predominantly neuronal protein that is normally bound to microtubules, where it acts to modulate neuronal and axonal stability. In humans, pathological forms of tau are implicated in a range of diseases that are collectively known as tauopathies. Kinases and phosphatases are responsible for maintaining the correct balance of tau phosphorylation to enable axons to be both stable and labile enough to function properly. In the early stages of tauopathies, this balance is interrupted leading to dissociation of tau from microtubules. This leaves microtubules prone to damage and phosphorylated tau prone to aggregation. Initially, phosphorylated tau forms oligomers, then fibrils, and ultimately neurofibrillary tangles (NFTs). It is widely accepted that the initial soluble oligomeric forms of tau are probably the most pathologically relevant species but there is relatively little quantitative information to explain exactly what their toxic effects are at the individual neuron level. Electrophysiology provides a valuable tool to help uncover the mechanisms of action of tau oligomers on synaptic transmission within single neurons. Understanding the concentration-, time-, and neuronal compartment-dependent actions of soluble tau oligomers on neuronal and synaptic properties are essential to understanding how best to counteract its effects and to develop effective treatment strategies. Here, we briefly discuss the standard approaches used to elucidate these actions, focusing on the advantages and shortcomings of the experimental procedures. Subsequently, we will describe a new approach that addresses specific challenges with the current methods, thus allowing real-time toxicity evaluation at the single-neuron level.

## Introduction

### Structure and Physiological Functions of Tau

**Figure 1:**
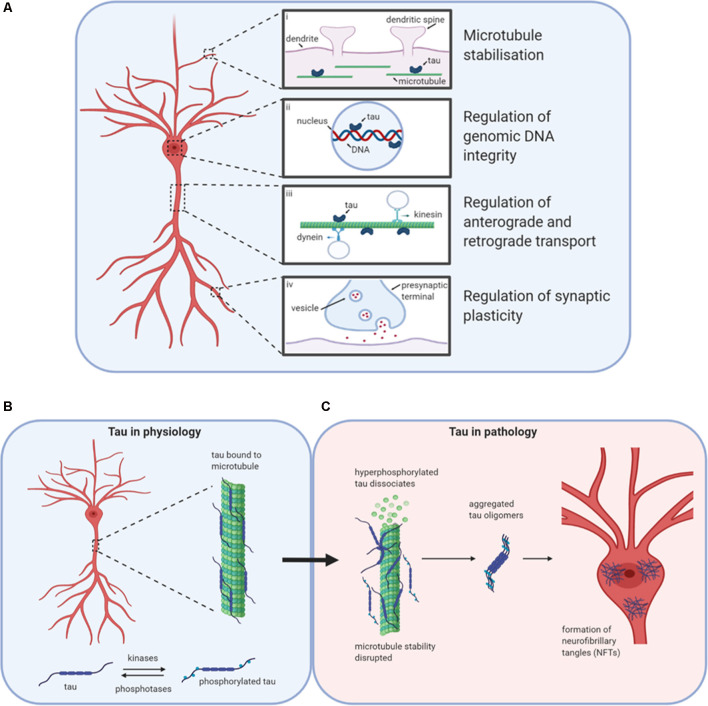
Tau in physiology and pathophysiology. Tau has many physiological roles in neurons **(A)**. (i) The most prominent function is in the modulation of microtubule stability and assembly. (ii) Tau also plays a role in the nucleus, potentially regulating the integrity of genomic DNA by binding to different chromosomal regions. (iii) The third role for tau in physiology is in the regulation of axonal transport by interfering with the binding of motor proteins kinesin and dynein to microtubules, thus altering the movement of cellular materials between the soma and axon. (iv) Finally, tau is believed to play important roles in the regulation of synaptic plasticity, for example by its contribution to the process of spine remodeling. Design concept from Wang and Mandelkow (2016). **(B)** Under physiological conditions, tau is bound to microtubules. The stability of microtubules is maintained by the balanced activities of kinases and phosphatases acting to regulate the binding of tau to the microtubules. **(C)** If the balance is altered in favor of increased kinase activity, then tau can become hyperphosphorylated and detach from the microtubules affecting stability and tau aggregation. Initially, monomers aggregate to form oligomers which are β-sheet-rich and are thought to be the smallest toxic species. Subsequently, these species aggregate further into protofibrils, fibrils, and finally into neurofibrillary tangles (NFTs). Created with BioRender.

Tau is encoded by the microtubule-associated protein tau (*MAPT*) gene. As a monomer, it is intrinsically disordered and lacks a significant secondary structure (Cleveland et al., 1977; Barghorn et al., 2004; Mukrasch et al., 2009; Mirbaha et al., 2018). Tau associates with microtubules and is key to maintaining cellular and axonal morphology as well as assisting in vesicle transport and trafficking (Drechsel et al., 1992; Brandt and Lee, 1993; Brandt et al., 1995; Tracy and Gan, 2018; Figure 1A). Tau binds to the labile domain of microtubules to modulate their stabilization and maintain free movement (Qiang et al., 2018). There are six isoforms of tau in the adult human brain, which are differentially expressed during development (Goedert et al., 1988, 1989; Goedert and Jakes, 2005). The acidic N-terminal region can have up to two inserts between amino acids 45 and 103 (generating 0N, 1N or 2N tau). The mid-region is rich in prolines, as well as serine and threonine residues, the phosphorylation of which have been linked to both physiological and pathophysiological processes (Stoothoff and Johnson, 2005; Hanger et al., 2007; Hampel et al., 2010; Wang et al., 2013). Towards the C-terminus, there are four pseudo-repeat domains (R1-R4), referred to as the microtubule-binding domains.

Tau can regulate axonal transport (Figure 1A), maintaining protein trafficking both from the axon to the soma (retrograde; Dixit et al., 2008) and from the soma to the axon (anterograde; Stamer et al., 2002). Tau acts as a competitor for dynein and kinesin binding sites on microtubules. It can also directly modulate kinesin binding *via* its N-terminus (Kanaan et al., 2011) or alter dynein binding by interactions with dynactin (Magnani et al., 2007). Although most tau is localized in axons, tau also plays a physiological role in dendrites, assisting with spine remodeling which is required for synaptic plasticity (Frandemiche et al., 2014; Regan et al., 2015; Figure 1A). Finally, tau has reported roles in the nucleus in human neuroblastoma cells, regulating the integrity of genomic DNA (Loomis et al., 1990; Figure 1A).

### Pathological Cascade Leading to Tangles

Tau function is modulated by post-translational modifications such as phosphorylation, acetylation, and ubiquitination (Beharry et al., 2014; Alonso et al., 2016). Finely regulated levels of phosphorylation (by kinases and phosphatases; Figure 1B) on specific residues contribute to the protein’s physiological functions (Stoothoff and Johnson, 2005). In disease states, regulation of the balance between phosphorylation and de-phosphorylation is lost, leading to increased abnormal (“hyper”) phosphorylation (Köpke et al., 1993). Hyperphosphorylated tau dissociates from microtubules and can sequester normal tau away from tubulin, disrupting microtubule function, and stability (Qiang et al., 2018). Aggregation-incompetent monomeric tau protein (Mirbaha et al., 2018) undergoes monomer-monomer interactions *via* disulfide bonding or hexapeptide motifs to drive a conformational change from an unfolded, disordered structure to a new structured conformation that is rich in beta-sheets and aggregation-competent (Friedhoff et al., 1998; Congdon et al., 2008). This marks the beginning of the pathological cascade starting with the aggregation to oligomeric structures, through to fibrils and then to neurofibrillary tangles (NFTs; Avila et al., 2006; Maeda et al., 2006; Figure 1C). NFTs were assumed to be the toxic species since their presence is characteristic of brain tau pathology (Braak and Braak, 1991). However, soluble tau oligomers formed much earlier in the aggregation process may be the primary drivers of toxicity (Lee et al., 2001; Wittman et al., 2001; Tanemura et al., 2002; Tatebayashi et al., 2002; Andorfer et al., 2003; Spires et al., 2006; Yoshiyama et al., 2007; Cowan et al., 2010). Reports that some toxic forms of tau with specific pathological mutations aggregate into oligomers but do not form fibrils further support this theory (Lasagna-Reeves et al., 2010, 2014; Karikari et al., 2019a). In cell culture experiments, tau oligomers applied to media were taken up into the nuclei of human neuroblastoma cells and stem cell-derived cortical neurons (Usenovic et al., 2015; Wegmann et al., 2016; Evans et al., 2018; Karikari et al., 2019a; Puangmalai et al., 2020). In humans and transgenic mice models, phospho-tau impairs nuclear transport by interacting with the nuclear pore complex and disrupting its activities (Eftekharzadeh et al., 2018). Toxic tau oligomers are transferred between neurons by extracellular release, then uptake by neighboring neurons where it induces the aggregation of endogenous tau and possibly other proteins (Walsh and Selkoe, 2016; Gibbons et al., 2019). This process has been studied extensively in cell and transgenic animal models (Clavaguera et al., 2009; Guo and Lee, 2011; Iba et al., 2013; Boluda et al., 2015; Usenovic et al., 2015; Wegmann et al., 2016; Narasimhan et al., 2017; Evans et al., 2018; Karikari et al., 2019b; He et al., 2020; Puangmalai et al., 2020) and is believed to be regulated by the low-density lipoprotein receptor-related protein 1 (Rauch et al., 2020).

### Primary and Secondary Tauopathies

Tauopathies represent a group of diseases in which neuronal dysfunction and loss are primarily governed by the presence of abnormal tau protein. Tauopathies have varying histopathology and clinical presentations and can often be distinguished by the isoforms and ultrastructure of the tau aggregates (Dickson et al., 2011; Rademakers et al., 2012). Tauopathies can be classified broadly by whether tau aggregates are the sole primary pathological feature. The most common tauopathy, Alzheimer’s disease (AD), is a secondary tauopathy due to the presence of other contributory pathological factors such as extracellular amyloid-beta plaques (Jack et al., 2013). The presence of amyloid-beta aggregates and phosphorylated tau aggregates (NFTs) are used for pathological confirmation of AD diagnosis at *post mortem* (Guillozet et al., 2003). In the human Alzheimer brain, tau aggregates spread stereotypically following the pathological staging of tau pathology proposed by Braak and Braak (Braak and Braak, 1991), as confirmed recently by neuroimaging studies (Vogel et al., 2020). Clinically, NFT accumulation is observed in the brains of AD patients and correlates well with disease progression (Wischik et al., 1988).

Tau also plays a role in mediating the pathogenesis of other oligomeric proteins such as amyloid-beta (Castillo-Carranza et al., 2015), alpha-synuclein (Castillo-Carranza et al., 2018; Teravskis et al., 2018) and prion protein (Reiniger et al., 2010; Corbett et al., 2020). One of the pioneering approaches to prepare tau oligomers *in vitro* induced tau aggregation with amyloid-beta or alpha-synuclein aggregates (Lasagna-Reeves et al., 2010). Similarly, tau oligomers have been shown to induce alpha-synuclein toxicity (Gerson et al., 2018). Furthermore, Puzzo et al. (2017) described a common mechanism for oligomeric amyloid-beta and tau toxicity involving the binding of the amyloid precursor protein (APP). The authors demonstrated APP-mediated defects in behavior and synaptic plasticity following the tau oligomer application. Oligomeric forms of tau are formed following mild traumatic brain injury (Bittar et al., 2019). Together, these results suggest that tau can act in concert with amyloid-beta (as shown in AD; Skillbäck et al., 2015) and alpha-synuclein (as in some cases of Parkinson’s disease; Goris et al., 2007 and Moussaud et al., 2014).

## The Pathological Relevance of Using Electrophysiology to Model Tau Functions in Physiology and Pathophysiology

Although the toxicity of tau oligomers has been studied extensively histopathologically in human and animal models, there is surprisingly little quantitative information describing their direct effects on neurons. Different studies employ a range of concentrations of tau oligomers, often higher than the physiological levels of the protein. Moreover, cells and animals are treated with tau oligomers for variable amounts of time. Consequently, it is unclear if cytotoxicity is dependent on tau oligomer concentration and/or duration of treatment. Since tau oligomers mediate cytotoxicity partly by altering synaptic and electrophysiological properties, it is important to document their concentration-, time- and structure-dependent actions on neurons. Electrophysiological techniques can provide direct measures of neuronal function and highlight the direct implications of aggregated tau for circuit function. The majority of studies have been carried out using *in vitro* rodent models. Recording from acutely isolated brain slices is a highly effective method as it allows much greater control, in terms of tau concentration and pharmacological manipulation than *in vivo* measurements, while retaining the cell architecture and local synaptic morphology.

### Electrophysiological Techniques: A Brief Overview

**Figure 2:**
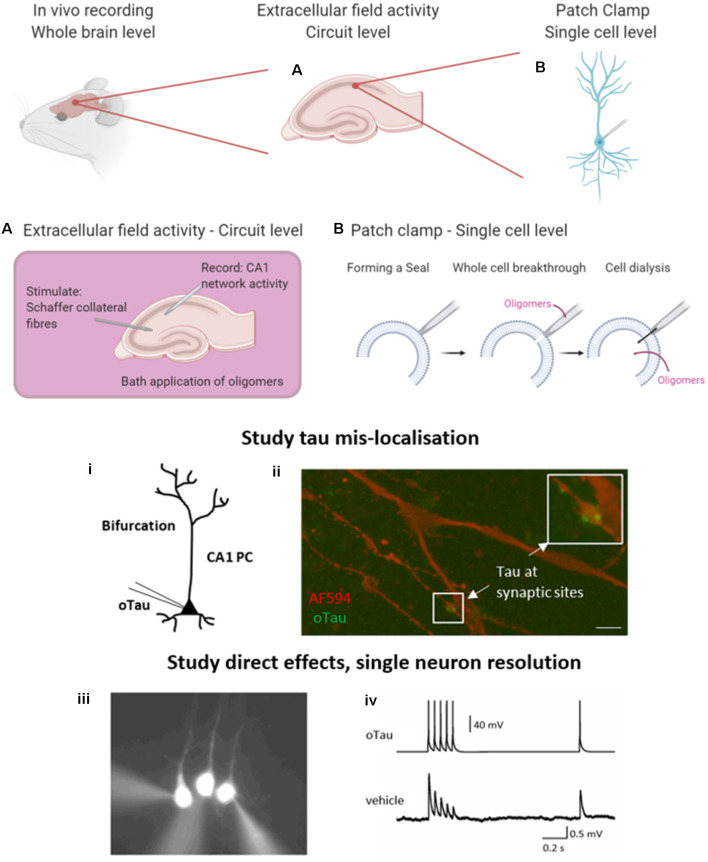
Electrophysiological approaches to measuring neuronal function. A brief overview of the common electrophysiological approaches used to evaluate tau oligomer function. *in vivo* experiments give the greatest level of translatability to the research as the whole brain remains intact. However, *in vitro* experiments permit much greater control over oligomer concentration and pharmacological manipulation. **(A)** A schematic illustration to demonstrate extracellular field recording in the CA1 region of the hippocampus. A stimulating electrode is placed in the Schaffer collateral fiber region and a recording electrode in the CA1 region where they synapse onto pyramidal cells, to record excitatory postsynaptic potentials (EPSPs). This technique allows for the study of local circuit activity. **(B)** The whole-cell patch-clamp allows for the parameterization of neuronal function on a single-cell level. By forming a high resistance seal between the pipette and the membrane of the target cell, then breaking through (with negative pressure), the contents of the patch pipette will dialyze into the cell. (i) This technique can be used to deliver proteins and peptides of known concentration and defined structure directly into target cells whilst simultaneously making current and voltage measurements in a network that is free from pathology. (ii) The whole-cell patch-clamp permits the study of tau mislocalization, with fluorescently labeled tau diffusing to synaptic structures above the dendritic bifurcation. Tau oligomers can be targeted to pre or postsynaptic neurons during multiple whole-cell recordings. (iii) An example where 3 layer V pyramidal cells were simultaneously recorded. The neuron on the left has tau oligomers introduced from the patch pipette whereas the other two cells receive a vehicle. (iv) Example traces from presynaptic neurons (tau oligomer) showing action potential firing pattern to induce EPSPs in postsynaptic neurons (vehicle). Created in part with BioRender. Reproduced in part using content previously published in Hill et al. (2019); eNeuro on a CC-BY 4.0 license.

Here, we briefly introduce two commonly used *in vitro* electrophysiological techniques that enable the study of tau oligomer-mediated effects on neuronal function. Recording extracellular field responses allows an overview at the circuit level and permits the understanding of local network dysfunction following the bath perfusion of oligomers or in transgenic models. Given that tau oligomers can disrupt synaptic transmission and plasticity, this is a valuable tool to evaluate effects on connectivity. Field recordings in the hippocampus are most commonly performed by stimulating the Schaffer collateral fibers and recording excitatory postsynaptic potentials (EPSPs) in the CA1 region where they make synapses onto pyramidal cells (Figure 2A). Details of how to perform extracellular field recordings are outlined by Zhang et al. (2014). Field recordings are useful as they sample many synapses and thus provide an average response. They can also be used to record action potential firing when the circuit is activated by excitant solutions.

The second technique permits a more targeted approach and the evaluation of effects at a single neuron level. The whole-cell patch-clamp recording is a well-established technique (originally developed by Neher and Sakmann, 1976), whereby a high resistance seal is formed between the tip of the recording pipette and the membrane of the target cell. Additional negative pressure ruptures the membrane (termed whole-cell breakthrough) and the contents of the patch pipette dialyze into the target neuron whilst allowing simultaneous current and voltage measurements from the recorded cell (Figure 2B). Details of how to perform whole-cell patch-clamp are discussed in Van Hook and Thoreson (2014). This method provides high-resolution measurements from single neurons including sub-threshold information (such as changes in membrane potential and input resistance) which is not obtainable from extracellular field recordings. This method can be used with various tau delivery approaches, including transgenic models, extracellular bath perfusion, or delivery *via* the patch pipette.

## Conventional Methods to Study Pathology: Lessons and Limitations

### Transgenic Models

A classic approach to study physiological tau function is to generate transgenic mouse models, for example by the genetic manipulation of the *MAPT* gene. This has been done in a variety of transgenic lines with phenotypic effects being compared with wild-type littermates. These approaches have greatly furthered our understanding of tau, however, there are limitations. Germline knockouts can result in compensation from other microtubule-associated proteins, which can complicate the interpretation of the data. For example, in *MAPT*^−/−^ mouse models there are limited functional effects, as other axonal proteins such as microtubule-associated protein 1A (MAP1A) can compensate to perform the microtubule-stabilization functions of tau (Harada et al., 1994; Dawson et al., 2001; Tucker et al., 2001; Fujio et al., 2007).

Most transgenic lines for the study of AD work by promoting either the aggregation or overexpression of tau, amyloid-beta (or its parent molecule, the APP) or a combination to replicate the protein species implicated in disease pathology. Overexpression of human tau in rodent models can result in synaptic and behavioral dysfunction as well as the neuronal loss (Andorfer et al., 2005; Polydoro et al., 2009), mediated by smaller aggregates including oligomers (Lee et al., 2001; Tanemura et al., 2002; Tatebayashi et al., 2002; Wittman et al., 2001; Andorfer et al., 2003; Spires et al., 2006; Yoshiyama et al., 2007; Cowan et al., 2010). A challenge with using electrophysiology to study overexpression lines is that whilst specific observations may be associated with the presence or absence of NFTs and other defined late-stage aggregates, they cannot provide an understanding of the exact form(s) of tau that is responsible. This introduces variation into the electrophysiology data and makes equating changes in neuronal properties with tau aggregate structure and concentration difficult.

Even in transgenic animals that are believed to preferably accumulate soluble tau oligomers, an opportunity to study the toxic potentials of oligomers of different sizes and forms would be highly valuable in answering several outstanding questions, including: (i) which forms of tau oligomers mediate most of the toxicity?; (ii) is aggregation the mediator of tau electrophysiological toxicity?; (iii) how does the presence of tau oligomers in an individual neuron affect its synaptic connections with neighboring neurons? and (iv) are these effects pre- or post-synaptic? In these transgenic models, it is presently not possible to quantify the form(s) of tau oligomers present within each recorded cell, limiting direct analysis of disease-relevant toxicity.

### Extracellular Application to Culture Models or to *in vitro* Slices

Another approach is to deliver oligomeric tau to *in vitro* brain slices by perfusion or to inject them *in vivo*. These studies describe tau-mediated synaptic, mitochondrial, and memory impairments (Lasagna-Reeves et al., 2012; Fá et al., 2016; Ondrejcak et al., 2018; Acquarone et al., 2019). An advantage of this approach over transgenic models is that these methods can facilitate both the study of recombinant preparations of tau oligomers and of tau oligomer preparations that have been isolated from human brains, for example from patients suffering from tau pathology (Lasagna-Reeves et al., 2012). This enhances the translatability of findings. One limitation is that the observed effects cannot be attributed to tau oligomers being intracellular, as the applied proteins may be acting extracellularly or indirectly through other cells, such as glia and astrocytes. It is also unclear how much oligomeric tau has been taken up into neurons, which may vary considerably from cell to cell. Although *in vivo* studies provide the advantage of applying tau oligomers within a living system, any effects observed cannot be attributed to specific forms or concentrations of tau oligomers. A protocol that can assess tau oligomer effects on neuronal function in a structurally- and concentration-defined manner would greatly assist in clarifying the outstanding questions listed above.

### Pathologically Relevant Functions of Tau Oligomers Identified Using Electrophysiology

Electrophysiological measurements have uncovered several pathophysiological mechanisms mediated by tau oligomers, including altering the intrinsic excitability of pyramidal neurons and modulating both short- and long-term plasticity (Hoover et al., 2010; Rocher et al., 2010; Lasagna-Reeves et al., 2012; Puzzo et al., 2017; Tamagnini et al., 2017; Mondragón-Rodríguez et al., 2018). In recent years, several new pathological roles for tau have been described—particularly regarding its ability to induce synaptic dysfunction. Zhou et al. (2017) suggest that this occurs as a result of tau mis-localizing to the presynaptic terminals and binding to synaptic vesicles. Through actin crosslinking, it reduces their motility resulting in reduced release rate. This is a clear pre-synaptic mechanism of dysfunction, but post-synaptic mechanisms have also been reported. For example, administrating intracerebroventricular injections of tau oligomers that were isolated from AD patient brains inhibited long-term potentiation (LTP), a form of synaptic plasticity thought to underlie memory formation (Ondrejcak et al., 2018).

## A New Approach to Studying Oligomeric Tau Pathology

Given the limitations of previous approaches, Kaufmann et al. (2016) described an approach utilizing structurally defined, concentration-controlled preparations of oligomers. Here, alpha-synuclein aggregates were introduced through the patch-pipette during whole-cell patch-clamp recording from cortical neurons *in vitro*, and the subsequent electrophysiological changes measured. This approach has since been implemented by other groups to study alpha-synuclein pathology (Wu et al., 2019) and has been extended by Hill et al. (2019) to examine tau oligomers. The authors demonstrated that oligomeric tau (prepared as described in Karikari et al., 2017, 2019a) can induce significant changes to action potential dynamics, disrupt basal synaptic transmission and inhibit synaptic plasticity on a 40-min time scale (Hill et al., 2019; Figure 2B) despite the concentration being significantly lower (44–666 nM) than the physiological concentration of tau (2 μM in neurons, Avila, 2010). Tau oligomers diffused from the site of introduction (soma) to synaptic sites during the 40-min recording (Hill et al., 2019). At presynaptic sites, tau oligomers induced the run-down of unitary excitatory postsynaptic potentials (EPSPs), which was associated with increased short-term depression. In contrast, the introduction of tau oligomers into postsynaptic neurons did not affect basal synaptic transmission but markedly impaired the induction of LTP (Hill et al., 2019). The introduction of equivalent concentrations of tau monomers did not affect neuronal or synaptic properties.

This single-neuron approach avoids a number of the limitations of previous electrophysiological approaches. It allows one cell to be targeted in a neural network that is free from tau pathology, allowing direct time- and concentration-dependent effects of tau oligomers to be measured. The amount of tau required for the experiments is small and if labeled, the introduced tau oligomers can be tracked within the cell (Figure 2B). Upon whole-cell breakthrough, each cell acts as its control, and changes to electrophysiological parameters can be monitored over time from this baseline. Tau oligomers can be targeted into pre- or postsynaptic cells so that separate effects on synaptic transmission or plasticity can be uncovered. This provides an unparalleled level of mechanistic detail.

## Discussion

Tau plays an indisputable role in several primary and secondary tauopathies, therefore anti-tau immunotherapy is an attractive target to slow down the progression or treat these diseases. Immunotherapies will target different epitopes of tau and therefore will alter its function in different ways. It is likely that, if immunotherapy is successful for one tauopathy, that same one form of immunotherapy may likely not be sufficient to work across all tauopathies and so a targeted approach will be needed (Sigurdsson, 2018). For example, “Gosuranemab” (Biogen, 2019) was unsuccessful at phase 2 for patients with progressive supranuclear palsy but is also now being tested in a phase 2 trial in AD. Basic science underpins the understanding of the mechanisms of tau mediated degeneration. This needs to guide the development of potential treatment strategies, to facilitate their timely success. Traditional electrophysiological approaches have described pathophysiological roles for oligomeric tau at synapses, particularly in disrupting synaptic transmission and plasticity (Lasagna-Reeves et al., 2012; Fá et al., 2016; Puzzo et al., 2017; Ondrejcak et al., 2018). Direct introduction into neurons has provided further detail into the different sites of action for the oligomers at pre- and post-synapses. It has also allowed the evaluation of the earliest changes to cellular electrophysiological properties (Hill et al., 2019). The remaining questions lie in further understanding of how tau oligomers are mediating these changes. Once this is known, better-targeted treatments can be developed. The earlier the stage of pathology that treatment can be initiated, the greater chance of success.

## Author Contributions

EH, MW, KM, and TKK wrote the manuscript.

## Conflict of Interest

The authors declare that the research was conducted in the absence of any commercial or financial relationships that could be construed as a potential conflict of interest.
